# Body integrity identity disorder using augmented reality: a symptom reduction study

**DOI:** 10.1136/bcr-2020-238554

**Published:** 2021-01-11

**Authors:** Collin Turbyne, Pelle de Koning, Jasper Zantvoord, Damiaan Denys

**Affiliations:** Psychiatry, Academic Medical Center, Amsterdam, Noord-Holland, The Netherlands

**Keywords:** psychiatry, virtual rehabilitation, ethics

## Abstract

Body integrity identity disorder (BIID) is a rare condition characterised by a discrepancy between specific areas of an individual’s perceived body image and body schema which causes the individual to disassociate those physical areas of their body from their internal representation. There are currently no efficacious, ethically unambiguous means for achieving long-lasting symptom reductions. In the case we present, two patients with BIID underwent an augmented reality (AR)-based simulation that virtually amputated their alienated limbs, allowing them to experience their ideal selves. During the exposure, both patients reported reductions in BIID-related complaints. These preliminary results suggest the existence of a possible therapeutic and diagnostic potential that AR possesses, which warrants further consideration within clinical healthcare settings.

## Background

Body integrity identity disorder (BIID) is a rare and secretive condition characterised by the feeling of a lack of bodily ownership over a certain healthy limb or limbs, which subsequently causes patients to report a desire to obtain an amputation in order to appear as their ideal self. Due to the taboo nature surrounding this condition, most individuals do not electively present their symptoms to medical professionals and are instead found on internet forums which are mostly private. This current state of affairs makes it especially difficult to provide an accurate assessment of the prevalence of BIID. Individuals with this condition often speak of having an ideal self, which refers to the physical appearance that they feel best reflects their true body schema. It is common that patients are very specific regarding the position of the feeling of undesirability of the limb. The primary motivation for these individuals to seek an amputation is to feel complete or to feel satisfied inside, with sexual motivations often being secondary.^1^ The onset of these desires is often seen in early childhood,^1 2^ with no evidence of them being influenced by perceived ugliness, pain, conversion or psychosis.^1^ The aetiological framework of this disorder is as complex as it is unresolved; it has been approached from both psychiatric^1–5^ and neuroscientific^6–10^ standpoints yet still remains unclassified in the *Diagnostic and Statistical Manual of Mental Disorders*. The inability to establish a clear consensus on the diagnostic criterion of this disorder ultimately stems from the dearth of studies and diagnostic tools currently available. As such, no standard psychopharmacological or psychotherapeutic treatments for this disorder have been devised. Though controversial, the only method that has been reported to result in definite symptom improvement is to surgically remove the alienated limbs^11^; however, this approach has been ardently contested with arguments being made in favour for^12 13^ and against^14 15^ doing so. While this approach has not been adopted into any known clinical treatment strategies, it has not deterred these individuals from attempting to perform self-amputations in an effort to rid themselves of their alienated limbs.^16 17^ Other, more common, reported behavioural sequelae include privately simulating their desired appearance, that is, limb binding/limb folding or the use of a wheelchair/crutches/prosthetics, in an attempt to relieve their symptoms.^4 16 17^

## Case presentation

Two adult male patients were recruited from a patient group located at the Amsterdam Universitair Medische Centra (location Academic Medical Center (AMC), Amsterdam, Netherlands). Both patients were diagnosed with BIID with a primary motivation to feel complete or satisfied inside by a certified psychiatrist working in the outpatient clinic who is specialised in body image-related disorders. Neither of the patients had any relevant comorbid diagnoses. One of the patients desires a single below-the-knee amputation on the right leg (patient 1), while the other desires a double above-the-knee amputation (patient 2).

Patient 1 reported that the onset of his desires began in early childhood after seeing another child in a wheelchair with a stump amputation. His persistent desires to feel complete inside led him to feeling alone and depressed throughout his childhood and into adulthood. He currently simulates his ideal self by seating himself in a wheelchair and tying his leg down to the chair in a way where his limb folds, creating the appearance of a stump. He simulates his ideal self as often as possible during the day but is only able to perform a simulation for around 1 hour at a time due to his alienated limb becoming temporarily paraesthetic. He has, and still does, consider self-amputation; however, he has never made an attempt due to recognising the danger associated with doing so.

Patient 2 similarly reported that the onset of his desires began in his early childhood after meeting a family friend with a one-sided lower leg amputation. As a child, he simulated his desired appearance through limb binding without having any understanding that others had similar desires to his own. Only after the advent of the internet did he discover the extent of his own desires, both socially and clinically. He has also always considered self-amputation, but has never taken any concrete actions to accomplish this.

## Investigations

At the time of the current investigation, patient 1 had previously sought medical treatment elsewhere from a physiatrist, an orthopaedic surgeon, and a professor who is specialised in amputations and complex regional pain syndrome with regard to seeking an amputation for his alienated limb. He was never prescribed any medications to help treat his BIID-related complaints. He previously received hypnosis therapy; however, it proved unsuccessful.

Patient 2 has never sought medical treatment for his BIID, even though his primary physician has been made aware of his condition. Therefore, he has never been prescribed any medication nor has he undergone any form of psychotherapy. Rather than seeking medical attention, the patient has sought to self-manage his BIID by trying to adapt it into his life, although he still struggles with being able to do so.

Neither patient has any family history of BIID or body image-related disorders.

## Treatment

In the present study, we investigated the effects of virtual amputation in non-amputated patients in order to determine whether or not this could influence the way they experienced their BIID. We accomplished this by administering an augmented reality telepresence to the ideal-self (ART-IS). Augmented reality (AR) is one particular medium within a larger spectrum of other emerging technologies that are collectively referred to as virtuality technologies. Currently, the two most prevalent virtuality technologies are AR and virtual reality (VR). AR infuses or omits features from an individual’s real environment with digital content in a way that gives the individual an impression that these modifications exist as natural features of their real environment. AR content can be presented on a hand-held device or by using a head-mounted display (HMD), which is a specialised pair of non-transparent glasses that display the virtual content to the user. VR, on the other hand, is an entirely digital experience whereby individuals are completely separated from their real environment and are immersed into a digitally created virtual environment. VR content is necessarily presented on an HMD. While no previous research has specifically investigated the use of virtuality technologies to alter body ownership in patients with BIID, VR has been previously used in different ways to alter the sense of body ownership in healthy populations.^18–20^ Aside from their amputation wishes, patients with BIID are otherwise considered healthy and clinically unremarkable.^1 4^

**Figure 1:**
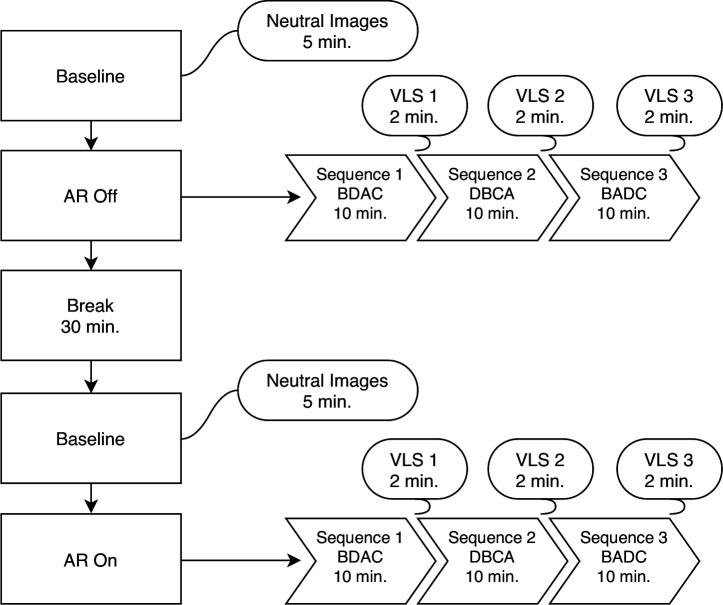
A complete overview of the procedure. AR, augmented reality; VLS, verbal Likert scale

We assessed the efficacy of ART-IS by performing and comparing symptom provocations in two different tasks, AR on and AR off (figure 1), using a repeated measures within-subjects design. The intertask interval was set to 30 min in order to help reduce the possibility of simulation sickness. In each task, we provoked the patients’ BIID by applying electrical stimulations over both the alienated and non-alienated regions of their limbs (energy light). The advantages of adopting this approach for stimulation are twofold; the intensity level of the stimulus can be administered consistently and the intensity levels are highly scalable, together yielding a high degree of accuracy and control during stimulus presentation. Before the experiment began, we adjusted the individual current intensities from each electrode on an individual-to-individual basis so that they were experienced as being moderately uncomfortable. This was determined by starting at the lowest intensity level and gradually increasing the intensity of the current until the individual reported a moderate level of discomfort. Moreover, before the patients began either task, we presented a series of neutral images in order to establish a baseline for our analysis. We assembled a unique set of neutral images for each baseline that consisted of five different nature scenes taken from a validated database^21^ and adapted them for use in an HMD. We administered these images at a rate of 1 min per image, which allowed the patients ample time to view each image.

Each task consisted of three sequences. Each sequence contained four blocks, each of which represented a different anatomical region. The blocks were presented pseudorandomly, such that each block could not be repeated back-to-back across or within sequences. The anatomical regions were (A) above the left knee, (B) below the left knee, (C) above the right knee and (D) below the right knee. Within each block, we administered six stimuli, each of which had a duration of 0.2 ms. The interstimulus interval was set to 20 s, which allowed us to obtain a clear and analysable physiological signal. The intersequence interval was set to 2 min in order to allow us a sufficient amount of time to administer a verbal Likert scale (VLS), which assessed the patients’ subjective experience of the symptom provocation. Each VLS consisted of five questions with answers ranging from 1 to 5: 1 indicated not at all; 2 indicated slightly; 3 indicated moderately; 4 indicated substantially; and 5 indicated extremely. These questions asked (1) how tense they were, (2) how much their BIID symptoms were disturbed or effected in the moment, (3) how much physical discomfort they were in, (4) how fearful they were and (5) how angry they were. We further evaluated the patients’ experiences by measuring heart rate (HR), respiratory sinus arrhythmia (RSA) and pre-ejection period (PEP) to assess autonomic nervous system activity. HR is an indicator of combined parasympathetic and sympathetic control.^22^ RSA is a naturally occurring variation in HR during a respiration cycle and has been shown to be a reliable way to assess parasympathetic control.^23^ PEP is the time interval between electrical stimulation of the ventricles and the opening of the aortic valves, which measures sympathetic control of cardiac activity.^24 25^ We measured these three physiological indicators by means of electrocardiography and impedance cardiography by using an ambulatory physiological recording device (Vrije Universiteit ambulatory monitoring system). We adhered to the operational use of the device according to the manual.^26^

**Figure 2:**
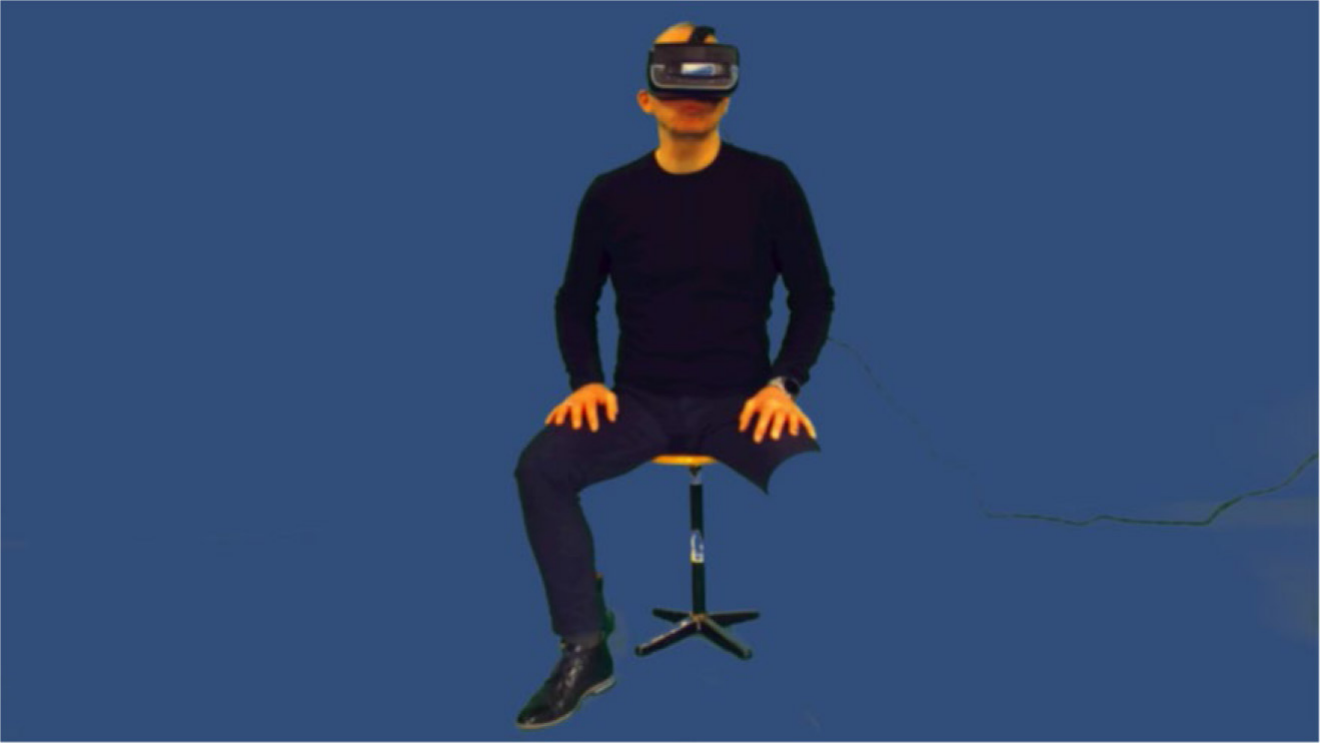
A raw image taken of a staff member demonstrating ART-IS with the AR function turned on. AR, augmented reality; ART-IS, augmented reality telepresence to the ideal self.

ART-IS was presented on an HMD (Oculus Rift DK2) from a fixed third person perspective, which was created by a video feed from a stereoscopic camera (Zed Mini) (figure 2). In order to virtually amputate the patients’ alienated limbs, we applied an active chroma key through the use of software running in a game engine (Unity3D). We had the patients sit down on a chair that was positioned in front of an evenly lit green screen backdrop. We then fitted their alienated limbs with a green sock which allowed us to evenly blend the limbs into the background.

## Outcome and follow-up

During AR off and AR on, both patients indicated low VLS scores (1 and 2) for questions addressing whether they had any physical discomfort, how fearful and how angry they were.

Patient 1 reported a score of 4 when asked how much his BIID was disturbed during the last AR off sequence and the last two AR on sequences, compared with reporting a score of 1 for the other sequences during both AR off and AR on. Due to the nature of his scoring, he was asked to further clarify his scoring rationale while responding. He provided further insight into his answer by saying that it was currently affecting his BIID in ‘a positive and welcoming way’ unlike AR off. Moreover, several days after testing, he reported a reduction in his symptoms via unprompted follow-up emails.

Patient 2 reported a decrease of ‘how tense he was’ from the first sequence to the third sequence during AR off with a score of 3 and 2, respectively. During AR on, his response of a 5 during the first sequence reduced to a score of 3 during the third sequence. Similar to patient 1, he was asked to provide further clarification during his response. He described his high level of tension ‘positively’ by stating that it was ‘emotionally overwhelming to finally see himself like this’.

**Figure 3:**
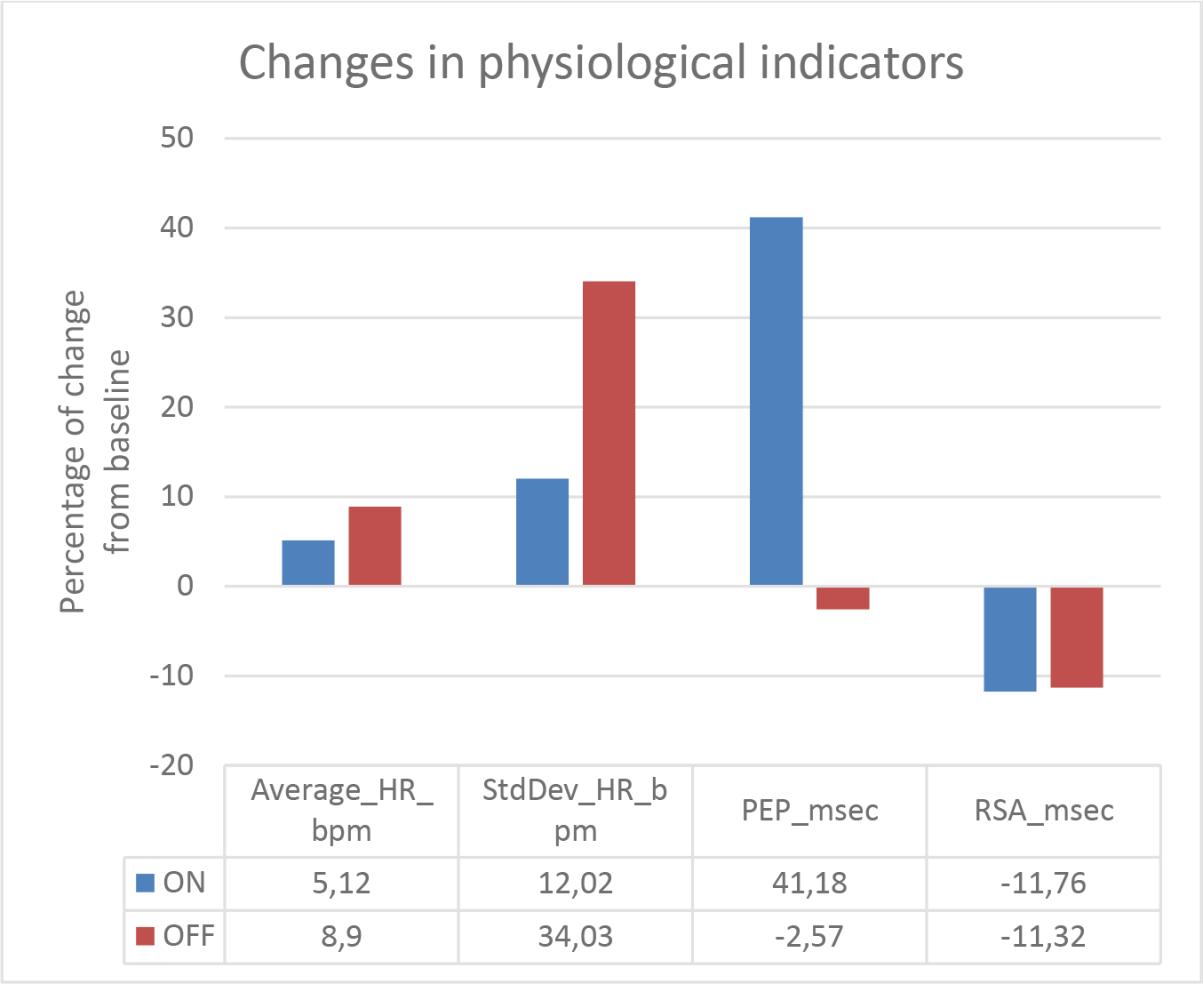
Changes in HR, PEP and RSA in response to electrical stimulation below the line of amputation for patient 1 during AR on and AR off. AR, augmented reality; HR, heart rate; PEP, pre-ejection period; RSA, respiratory sinus arrhythmia.

**Figure 4:**
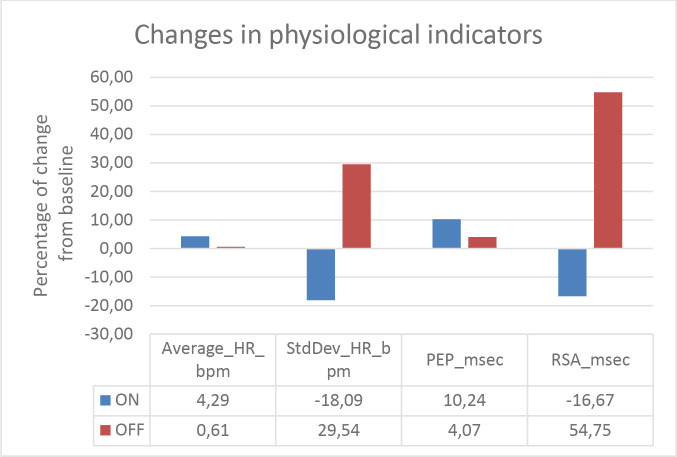
Changes in HR, PEP and RSA in response to electrical stimulation below the line of amputation for patient 2 during AR on and AR off. AR, augmented reality; HR, heart rate; PEP, pre-ejection period; RSA, respiratory sinus arrhythmia.

The primary, within-subjects, physiological outcome measures are depicted in figures 3 and 4 for patients 1 and 2, respectively. In patient 1, we found lower HR during AR on compared with AR off. For patient 2, we found higher HR during AR on compared with AR off. We found between-subjects overlap for the remaining indicators. For both patients, we found increased activity in PEP and decreased activity in RSA during AR on compared with AR off.

## Discussion

In this study, we investigated whether ART-IS influences the way BIID patients experience their alienated limbs. During testing, the self-report data from both patients indicated that ART-IS was well tolerated. This was confirmed directly after the exposure as both patients expressed the desire to experience ART-IS again in the future. When asked if the exposure increased their desire to obtain an amputation, both patients replied that it did not. Patient 1 added to this question by stating that this exposure validated his desires to obtain an amputation. Moreover, patient 1 also reported a reduction in his symptoms for several days after testing, indicating that there may have been a carry-over effect from the exposure. This could be interpreted as a treatment option rather than a mere carry-over effect. In both patients, we found increased activity of PEP and decreased activity of RSA during AR on compared with when it was off, indicating a physiological stress response. Our observed interactions between sympathetic and parasympathetic nervous system activity are in line with results from other studies that used the Trier Social Stress Task, which is a well-documented task that has been shown to reliably elicit psychological distress and a physiological stress response.^27^ In terms of clarifying the relation between PEP and RSA, recent preprinted research has suggested that SNS and PNS are reciprocally coupled with each other during rest and in response to an acute stressor.^28^

During data collection for patient 1, stimulation was applied incorrectly for the first two stimuli of the first B block in the AR on task by applying stimulation to D. This error was quickly identified and corrected during testing. Due to this, we decided that rather than excluding these data, we would instead reassign these data under the correct label.

One of the limitations surrounding the current research is that the VLS we administered during our exposure has not been used or validated. Because we are unaware of a validated questionnaire that assesses the effects of virtual amputation in non-amputated individuals, we resorted to creating our own. In order to better interpret the physiological signals of interest, we had to better understand what factors could be contributing to a potential stress response while administering uncomfortable stimuli. Therefore, our questions also reflected other, non-BIID-related, factors that would allow us to better explain potentially confounding variables. Therefore, future investigations should seek to establish and validate a standardised questionnaire that is suitable for assessing the effects of virtual amputation in non-amputated patients. Another limitation of this study was that we were restricted to observing individuals with the desire for an amputation of their lower extremities (ie, single or double leg). While lower extremity amputation wishes are common variants within this population,^1 4^ future research should also seek to include those with upper extremity amputation wishes (ie, fingers, hands and arms). Because ART-IS is a modular approach, it is not limited to any one specific variant of BIID or perspective of exposing the individual to their ideal self (ie, from the first or third person), which makes it suitable for a wide array of clinical investigations.

While more research is still needed, it is possible that this method may suffice as a stand-alone application that is able to gradually ameliorate BIID symptoms. Conversely, it could be used as an adjunct during neuromodulation therapy^29^ or for AR-based behavioural training that attempts to reintegrate the alienated limb.^30^ Future iterations of this method should also consider adapting this application for a mobile set up (eg, portable AR glasses) in order to study the longitudinal effects of exposure to the ideal self, which will ultimately determine the clinical utility of this method.

**Learning points**

- Emerging virtuality applications have the potential to act as a therapeutic adjunct to treatment for individuals with body integrity identity disorder (BIID).
- Emerging virtuality applications have the potential to enhance diagnostic insight into this medically and ethically complex disorder.
- Augmented reality telepresence to the ideal-self can be used for any variation of BIID in both clinical or at-home settings.
- Future iterations of this application should focus on the longitudinal effects of longer and more repetitive exposures to the ideal self in virtual environments.
